# Cross-Species Root Transcriptional Network Analysis Highlights Conserved Modules in Response to Nitrate between Maize and Sorghum

**DOI:** 10.3390/ijms21041445

**Published:** 2020-02-20

**Authors:** Hongyang Du, Lihua Ning, Bing He, Yuancong Wang, Min Ge, Jinyan Xu, Han Zhao

**Affiliations:** Jiangsu Provincial Key Laboratory of Agrobiology, Institute of Crop Germplasm and Biotechnology, Jiangsu Academy of Agricultural Sciences, Nanjing 210014, China; hongyangdu@foxmail.com (H.D.); NLH_2015@126.com (L.N.); hebing1980@aliyun.com (B.H.); wangyuancong@163.com (Y.W.); gemin8614@163.com (M.G.); xujinyan0610@126.com (J.X.)

**Keywords:** maize, sorghum, nitrate, cross-species, transcriptional network

## Abstract

Plants have evolved complex mechanisms to respond to the fluctuation of available nitrogen (N) in soil, but the genetic mechanisms underlying the N response in crops are not well-documented. In this study, we generated a time series of NO_3_^−^-mediated transcriptional profiles in roots of maize and sorghum, respectively. Using weighted gene co-expression network analysis, we identified modules of co-expressed genes that related to NO_3_^−^ treatments. A cross-species comparison revealed 22 conserved modules, of which four were related to hormone signaling, suggesting that hormones participate in the early nitrate response. Three other modules are composed of genes that are mainly upregulated by NO_3_^−^ and involved in nitrogen and carbohydrate metabolism, including *NRT*, *NIR*, *NIA*, *FNR*, and *G6PD2*. Two G2-like transcription factors (*ZmNIGT1* and *SbNIGT1*), induced by NO_3_^−^ stimulation, were identified as hub transcription factors (TFs) in the modules. Transient assays demonstrated that ZmNIGT1 and SbNIGT1 are transcriptional repressors. We identified the target genes of ZmNIGT1 by DNA affinity-purification sequencing (DAP-Seq) and found that they were significantly enriched in catalytic activity, including carbon, nitrogen, and other nutrient metabolism. A set of ZmNIGT1 targets encode transcription factors (ERF, ARF, and AGL) that are involved in hormone signaling and root development. We propose that ZmNIGT1 and SbNIGT1 are negative regulators of nitrate responses that play an important role in optimizing nutrition metabolism and root morphogenesis. Together with conserved N responsive modules, our study indicated that, to encounter N variation in soil, maize and sorghum have evolved an NO_3_^−^-regulatory network containing a set of conserved modules and transcription factors.

## 1. Introduction

Maize (*Zea mays*) is an important cereal crop that grows globally in various agro-ecological environments. It not only provides human food and high-quality vegetable oils but also a variety of livestock feed and industrial materials. Sorghum (*Sorghum bicolor*) is the fifth most globally cultivated cereal crop and an important source of fodder and biofuel. Nitrogen is the most demanding macronutrient in plants and critical for plant growth, particularly for the production in high-yielding crop systems. Maize and sorghum perform C4 photosynthesis, which makes them exhibit a marked biomass and yield response to nitrogen fertilizers [1,2].

The main nitrogen forms used by plants in soil are nitrate (NO_3_^−^) and ammonium (NH_4_^+^). Nitrate transporter (NRT) and ammonium transporter (AMT) mediate their absorption in roots, respectively. Nitrate is the main form of nitrogen uptake by arid plants. Except as the main source of inorganic nitrogen, nitrate also acts as a signaling molecule, which has received great attention in the regulation of lateral-root development [3]. The lack of natural soil nitrogen and the excessive fertilization of nitrogen have negatively affected plant growth and crop yield. As soil particles are not easily combined with nitrogen, crops can only absorb 30% to 50% of applied nitrogen fertilizers, while loss to the atmosphere (NH_3_ and N_2_O) or water (NO_3_^−^) causes environmental pollution [4]. Understanding the mechanism of nitrogen (N) regulatory networks in plants is a crucial work for the goal of improving nitrogen-use efficiency, which may contribute to sustainable agricultural production by diminishing the use of nitrogen fertilizers.

Primary nitrate response (PNR) refers to the reaction of NO_3_^−^-depleted plants to NO_3_^−^, where PNR induces a profound reorganization of genome-wide gene expression. Indeed, thousands of genes are affected by PNR, and the extent of this transcriptional reprogramming appears to be conserved between plant species [5]. *Arabidopsis*, a model plant, has received much attention for its nitrogen transport, sensing and response [6,7]. Transcription factors (TFs) are the main drivers of transcriptional networks, and a handful of TFs involved in nitrate perception/signaling have to date been identified in *Arabidopsis*, including ANR1 [8], NPL7 [9], NLP6 [10], NLP8 [11], NAC4 [12], bZIP1 [13], TCP20 [14], NIGT1/HRS1 [15], TGA1/TGA4 [16], and LBD37/38/39 [17]. However, much of nitrate perception and signaling in other plants has been uncovered, especially with those crops that depend on nitrogen fertilizers for high grain yields. The molecular mechanisms they use to regulate nitrate response remain to be illuminated. Considering the complexity of NO_3_^−^-regulatory networks, system-biology approaches that integrate bioinformatics and molecular biology have proven useful for underlying regulatory networks and for identifying nitrate-related transcription factors [18].

Co-expression network analysis, aimed at understanding global instead of individual genes, considers all samples together and establishes connections between genes based on the collective information. It is a powerful system approach to accelerate the elucidation of molecular mechanisms underlying important biological processes [19]. Weighted gene co-expression network analysis (WGCNA), one of the most useful gene co-expression network-based methods, describes the correlation patterns between genes across transcriptomic datasets [20]. It has been used in identifying functional modules and gene networks, in which each node represents a gene, and the connecting lines (edges) represent co-expression correlations. Hub nodes (genes) are those that show most connections in the networks, and they are expected to play an important role in biology processes.

Comparative genomics provides a powerful approach to exploit genetic variation, differential gene expression, and evolutionary dynamics across various species. Analysis of genes in a phylogenetic context allows us to better understand how complex biological processes are regulated at the molecular level. Synteny refers to the conservation of the type and relative order of genes between different species differentiated by the same ancestor type. It has proven that, compared to orthologous genes based on phylogenetic analysis but located at nonsyntenic locations, syntenic orthologous genes are more likely to share correlated expression patterns [21]. After diverging from sorghum, maize underwent whole-genome duplication ~12 million years ago, resulting in a mesotetraploid genome [22]. Although the size of the maize genome (B73, ~2.1 Gb) is more than twice that of sorghum (BTx623, ~0.8 Gb), the difference in the number of genes is not very large [23,24]. Comparing the expression profiles of series time points after cold treatment, a core set of genes involved in perceiving and responding to cold stress are subject to functionally constrained cold-responsive regulation across maize and sorghum [25].

Previously, several studies explored the transcriptional reprogramming of nitrogen (N)-response in maize [26,27,28] and sorghum [29,30]. However, limited time points after N stimulation were explored in these studies, and the dynamics of N-regulatory networks remained to be illuminated. Comprehensive and accurate insight into dynamic transcriptional reprogramming after NO_3_^−^ provoking is a key step in the system-level understanding of NO_3_^−^-regulatory networks. To go beyond earlier studies, we generated time-series transcriptome data on nitrate-responsive gene expression in maize and sorghum roots. We performed weighted gene co-expression network analysis (WGCNA) of RNA-Seq data and constructed conserved NO_3_^−^-regulatory networks in two species (Figure S1). In addition, we identified several hub transcription factors that may act as key regulators in response to nitrate. Collectively, our work illuminates how the application of cross-species transcriptional network analysis can improve our understanding of plant root nitrate response.

## 2. Result

### 2.1. Genome-Wide Expression Effects of NO_3_^−^ Treatments in Maize and Sorghum Root

In this study, we identified differentially expressed genes (DEGs) in each species by comparing gene expression data in NO_3_^−^ treatments to those subjected to Cl^¯^ treatments. This analysis yielded a set of 2920 DEGs in maize and 1975 DEGs in sorghum (Supplemental Data S1 and S2). Several known genes involved in nitrate absorption and assimilation were highly upregulated by NO_3_^−^ (Figure S2), including *NRT2*, *NAR2*/*NRT3.1*, *NIA1*, *NIR*, *GLN1*, *GLT1*, and *ASN1*. Several carbohydrate-metabolism-related genes were identified in the DEGs, including glucose-6-phosphate dehydrogenase 2 (*G6PD*) and 6-phosphogluconate dehydrogenase (*PGD3*). To support the RNA-Seq results, qRT-PCR validation was performed on chosen transcripts. Nine differentially expressed genes were selected for qRT-PCR analysis in maize and sorghum, respectively. This contained genes that are involved in nitrate assimilation (*NRT*, *NAR*, and *NIA*) and carbohydrate metabolism (*G6PD*). The gene expression trend of qRT-PCR data was in accordance with RNA-Seq data, confirming the high reliability of the RNA-Seq approach (Figures S3 and S4).

**Figure 1:**
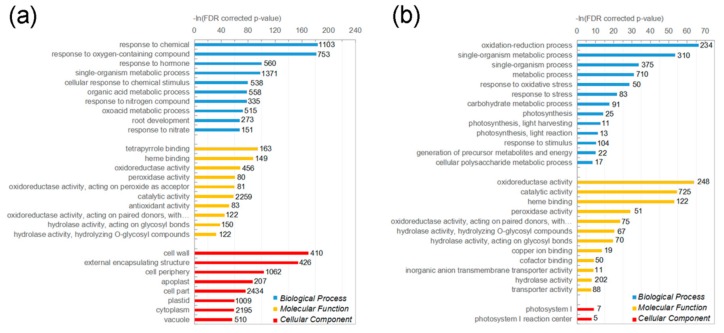
Gene Ontology (GO) classification of the differentially expressed genes (DEGs) identified in (**a**) maize and (**b**) sorghum. Number next to bar represents number of genes in GO term.

To understand the functions of those DEGs, we mapped them to Gene Ontology (GO) terms using agriGO 2.0. A total of 2631 maize DEGs were annotated by the GO database, and 849 significantly enriched GO terms were identified (Figure 1a, Supplemental Data S3). According to biological process, the DEGs mapped to four major GO terms: response to chemical (GO: 0042221), response to oxygen-containing compound (GO: 1901700), response to hormone (GO: 0009725) and single-organism metabolic process (GO: 0044710), accounting for 41.9%, 28.6%, 21.3%, and 52.1% of the annotated DEGs, respectively. In addition, 273 genes were mapped to root development (GO: 0009765). According to molecular function, the DEGs were mainly mapped to catalytic activity (GO: 0004553), accounting for 85.9% of the annotated DEGs. According to cellular component, the DEGs that mapped to cell periphery (GO: 0071944), cell part (GO: 0044464), plastid (GO: 0009536), and cytoplasm (GO: 0005737), constituted a high proportion under nitrate treatment.

When it comes to sorghum, 1202 DEGs were annotated, and 32 significantly enriched GO terms were identified (Figure 1b, Supplemental Data S3), which were much fewer than those of maize. According to biological process, the DEGs that mapped to oxidation-reduction process (GO: 0055114), single-organism metabolic process (GO: 0044710), single-organism process (GO: 0044699), and metabolic process (GO: 0008152) constituted a high proportion, accounting for 19.5%, 25.8%, 31.2%, and 59.0% of the annotated DEGs, respectively. According to molecular function, the DEGs were mainly mapped to catalytic activity (GO: 0003824) that was similar to that of maize. According to cellular component, there were no significant enriched GO terms. Compared to maize, the enriched GO terms from the biological process and molecular function that were identified in sorghum were easily found in maize, implying that maize and sorghum respond similarly to nitrate treatment.

### 2.2. Co-Expression Network Analysis Identified Modules That Responded to NO_3_^−^ in Maize and Sorghum

**Figure 2:**
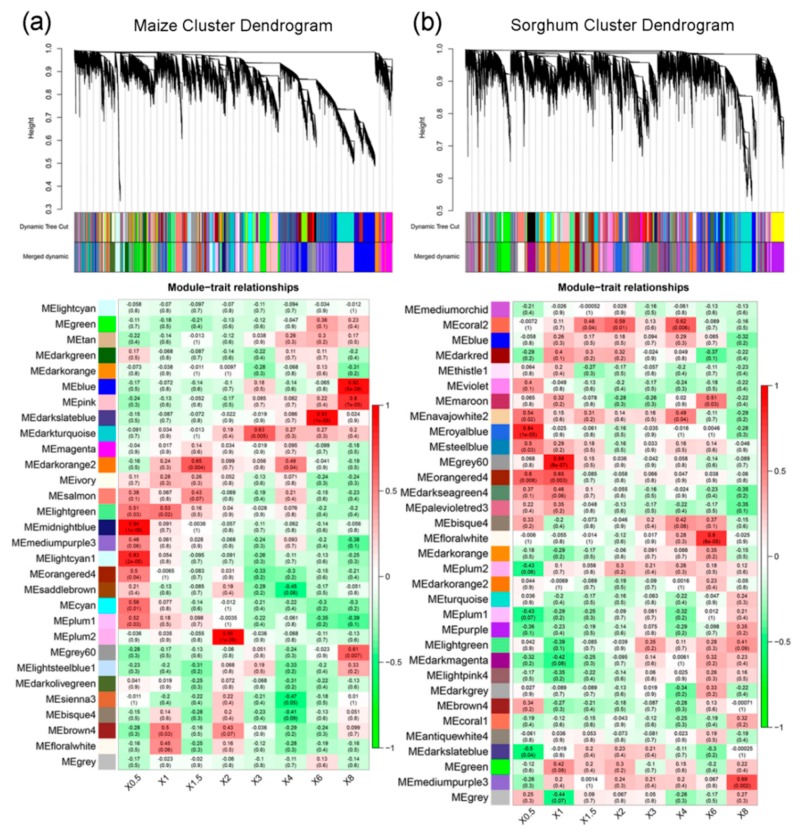
Weighted gene co-expression network analysis (WGCNA) based on gene expression matrix from (**a**) maize and (**b**) sorghum. Hierarchical cluster tree showing co-expression modules identified by WGCNA. Each tree leaf is one gene. Major tree branches constitute different modules labeled by different colors. Heatmap reporting correlations (r) and corresponding *p*-values (p) between selected modules and time indicators. Each row corresponds to a module. Each column corresponds to a specific time point. Color of each cell at row-column intersection indicates correlation coefficient between module and time stage.

To begin to decode the dynamic regulatory network, we used WGCNA to partition the set of genes into clusters of co-expression genes that share similar expression dynamics. Co-expression networks were constructed on the basis of pairwise correlations between genes in their common expression trends across all tissue samples. This analysis resulted in 30 modules in maize shown by dendrogram (Figure 2a, Supplemental Data S4), and gene numbers in these modules ranged from 62 (plum2) to 5711 (blue). The 30 module eigengenes for the 30 distinct modules were each correlated with a distinct stage due to the eigengenes’ time-specific expression profiles. Notably, thirteen of them showed to be significantly positively correlated (r > 0.5, *p* < 0.05) with NO_3_^−^ treatments (Figure 2a). To further identify modular features with biological roles related to NO_3_^−^ treatments, agriGO 2.0 was used to perform GO enrichment analysis (Supplemental Data S4). The midnightblue and lightgreen modules were significantly enriched in hormone-related GO terms. The darkturquoise module was significantly enriched in response to nitrate (GO:0010167, *p*-value: 1.8 × 10^−13^). The blue module was significantly enriched in root development (GO:0048364, *p*-value: 5.2 × 10^−21^). Taken together, these key modules suggested that nitrate not only induces a metabolic response but also triggers hormone-signal transduction and root development.

There were 33 distinct modules in sorghum (Figure 2b, Supplemental Data S5), and gene numbers in these modules ranged from 82 (coral2) to 2055 (darkorange). Notably, nine of them comprised genes that were significantly positively correlated (r > 0.5, *p* < 0.05) with NO_3_^−^ treatments (Figure 2b). We observed five modules that were correlated with NO_3_^−^ treatments at 0.5 or 1 h: navajowhite2, royalblue, steelblue, grey60, and orangered4, indicating putatively important roles for these modules in the early response to NO_3_^−^ treatments. We further characterized the differentially expressed genes in these five modules, and many genes that are directly involved in nitrate assimilation and carbohydrate metabolism were presented. GO enrichment analysis revealed that the enriched GO terms were indeed related to oxidation-reduction process (GO:0055114, *p*-value: 1.3 × 10^−6^), transmembrane transport (GO:0055085, *p*-value: 2.6 × 10^−6^), and single-organism metabolic process (GO:0044710, *p*-value: 7.7 × 10^−6^).

### 2.3. Cross-Species Transcriptional Network Analysis Identified Conserved Modules in Response to NO_3_^−^ in Maize and Sorghum

Since important biological processes are often evolutionarily conserved, we compared the co-expression networks of maize and sorghum to decipher the conserved regulatory networks that respond to nitrate. A total of 18,225 syntenic orthologous pairs were identified between 17,316 maize genes and 13,418 sorghum genes (Supplemental Data S6). The number of genes is different because, in some cases, sorghum genes have more than one syntenic orthologous in maize, while in other cases maize genes have more than one syntenic orthologous in sorghum. On the basis of orthologous gene pairs, we calculated the overlapped *p*-values of modules between maize and sorghum were calculated (Supplemental Data S7).

There were eleven maize modules that significantly overlapped with eleven sorghum modules (Table 1), of which five modules from maize (lightgreen, midnightblue, lightcyan1, darkturquoise, and pink) showed to be significantly positively correlated with NO_3_^−^ treatments (Figure 2a). These significantly overlapped modules may function conserved in response to nitrate. GO enrichment analysis revealed that the conserved modules from maize are associated with various biological processed (Table 1).

The midnightblue, salmon, and lightgreen modules of maize were conserved with the violet module of sorghum (Table 1). GO enrichment analysis revealed that three modules were all associated with hormone-mediated signaling (Figure S5), suggesting that hormone signaling participates in early nitrate response. These were 46 pairs of syntenic DEGs between the three modules and the violet module of sorghum (Table S4). Four pairs encode AP2/ERF transcription factors; six pairs encode JAZ proteins, which are inhibitors of jasmonic acid (JA) pathway. Two pairs encode fatty acid desaturase 8 (FAD8), which are involved in JAs biosynthetic process. Two pairs encode amidohydrolases, which contribute to jasmonoyl-isoleucine hormone turnover and generate 12-hydroxyjasmonic acid. *GRMZM2G120320* and *Sobic.004G065900* encode WRKY transcription factors that are homologous to *Arabidopsis* WRKY40, a key regulator in ABA signaling.

### 2.4. Nitrate Assimilation and Carbohydrate Metabolism Significantly Enriched in Conserved Modules

**Figure 3:**
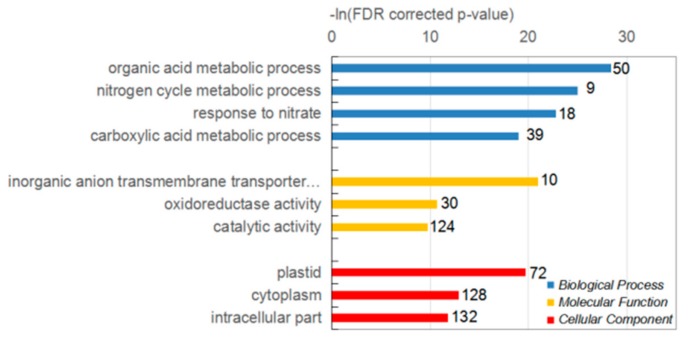
Gene Ontology (GO) classification of genes from darkturquoise module of maize. Number next to bar represents number of genes in GO term.

Among the conserved modules, the most significant ones were the darkturquoise module of maize and the grey60 and orangered4 modules of sorghum. These three modules showed significant positive correlation with NO_3_^−^ treatments (Figure 2a,b), and contained the most genes that directly involved in nitrogen and carbohydrate metabolism. In maize, 475 genes of the darkturquoise module were annotated by the GO database (Figure 3), enriched with GO terms like response to chemical (GO:0042221, *p*-value: 5.5 × 10^−21^), response to nitrate (GO:0010167, *p*-value: 1.8 × 10^−1^^3^), and response to organic substance (GO:0010033, *p*-value: 2.0 × 10^−1^^2^).

These were 24 pairs of syntenic DEGs between the darkturquoise module of maize, and the grey60 and orangered4 modules of sorghum (Table 2), including genes that are directly involved in nitrate assimilation (*FNR1*, *NIR1*, *NRT2.5*, and *NIA1*), and carbohydrate metabolism (*MDH*, *G6PD2*, *PGD3*, and *PGI1*). Fifty of the beat hit *Arabidopsis* genes of the 24 conserved pairs were upregulated by nitrate in at least ten independent experiments [31]. There were one pair of ferredoxin 3 (*FD3*), two pairs of *UPM1*, and two pair genes that encode oxidoreductase (2OG-Fe (II) oxygenase) in the syntenic orthologous list (Table 2). UPM1 is a key enzyme in the biosynthesis of sirohaem, prosthetic group of NIR, playing a crucial role in nitrate assimilation. *GRMZM2G016749* and *Sobic.006G064100* encode protein phosphatase 2C (PP2C) that may regulate the CLAVATA pathway. CLE-CLAVATA1 signaling has been proved to regulate the expansion of root systems in a nitrogen-dependent manner in *Arabidopsis* [32]. *GRMZM2G133684* and *Sobic.001G134800* encode integral membrane proteins of the HPP family; its homology in *Arabidopsis* (*At3g47980*) has been provided to be a component of the nitrite transport system of plastids [33]. *GRMZM2G173882* and *Sobic.002G016300* encoded G2-like transcription factors.

### 2.5. Identification of Hub Transcription Factors in Nitrate-Assimilation-Related Modules

**Figure 4:**
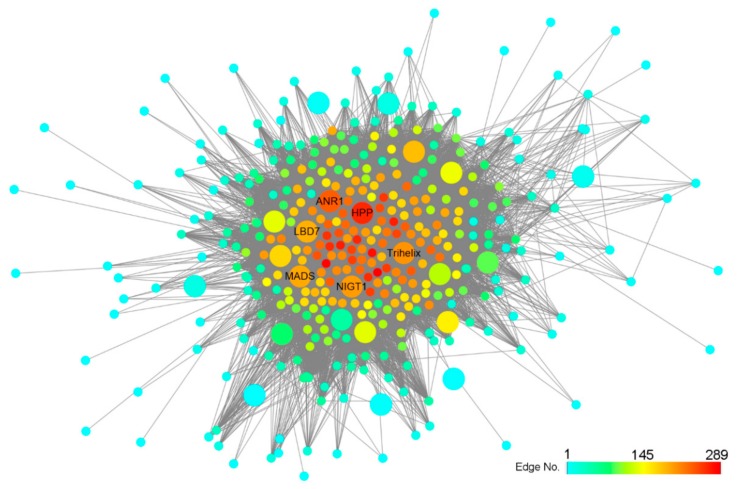
Graphical representation of darkturquoise module of maize. Genes from modules were extracted with weight threshold 0.1 of maize and exported to an edge file and node file for visualization by Cytoscape. Transcription factors and HPP shown by larger circles. HPP, *GRMZM2G133684*; ANR1, *GRMZM2G044408*; Trihelix, *GRMZM2G162840*; NIGT1, *GRMZM2G173882*; MADS, *GRMZM2G098986*; LBD7, *GRMZM2G017319*.

Module eigengenes (also called hub genes) are those that show the most connections in the network. The NO_3_^−^-regulatory network is highly complex, and transcription factors can act as potential regulators in controlling gene expression; hence, we identified the hub transcription factors as central genes in response to nitrate. In the network of the darkturquoise module of maize (Figure 4), 38 of the 550 genes encoded transcription factors, and the top-five putative hub TFs were *ZmANR1*, *ZmTrihelix*, *ZmNIGT1*, *ZmMADS,* and *ZmLBD7*. *ZmANR1* encodes a MADS-box transcription factor that was homologous to *Arabidopsis ANR1*, which was involved in nitrate-dependent signaling [8]. *ZmNIGT1* encodes a G2-like transcription factor that was homologous to *Arabidopsis NIGT1*, which has been proved to integrate nitrate and phosphate signals at the root tip [15]. *ZmLBD7* was homologous to *Arabidopsis LBD37*, which was also biologically validated affect nitrate response [17]. Strikingly, *ZmHPP* (*GRMZM2G133684*) ranked in the top positions of the network, implying that it has an important role in nitrate assimilation.

In the network of the grey60 module of sorghum (Figure S6A), 27 of the 587 genes encoded transcription factors, and the top-three putative hub TFs were *Sobic.001G314900* (TALE), *Sobic.002G247500* (EIL), and *Sobic.004G113700* (C3H). *Sobic.002G247500* was homologous to *Arabidopsis EIL3* that encodes a central transcriptional regulator of sulfur response and metabolism [34]. In the network of orangered4 module of sorghum (Figure S6B), fifteen of the 192 genes encoded transcription factors, and the top putative hub TFs were *Sobic.009G160000* (bHLH), *Sobic.005G064600* (NAC), *Sobic.001G328500* (LBD6), *Sobic.001G020200* (C2H2), and *Sobic.002G016300* (NIGT1). *Sobic.009G160000* was homologous to *Arabidopsis ILR3*, which modulates multiple stress responses [35]. *Sobic.002G081300* encodes an ERF transcription factor that was homologous to *Arabidopsis* CRF4, which was validated to regulate a significant number of genes in the dynamic N response [36]. Together with the hub TFs of maize, the transcription factors from LBD and G2-like families might function conserved in maize and sorghum and act as master regulators in response to nitrate.

### 2.6. ZmNIGT1 and SbNIGT1 Encode G2-Like Transcription Factors with Transcriptional Inhibitory Activity

**Figure 5:**
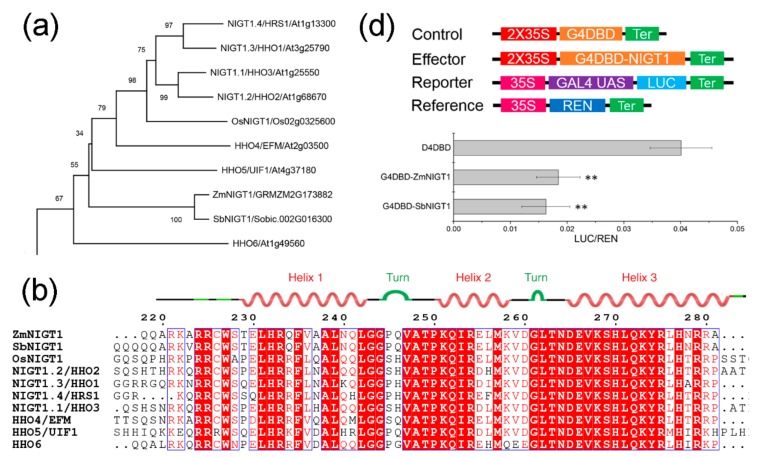

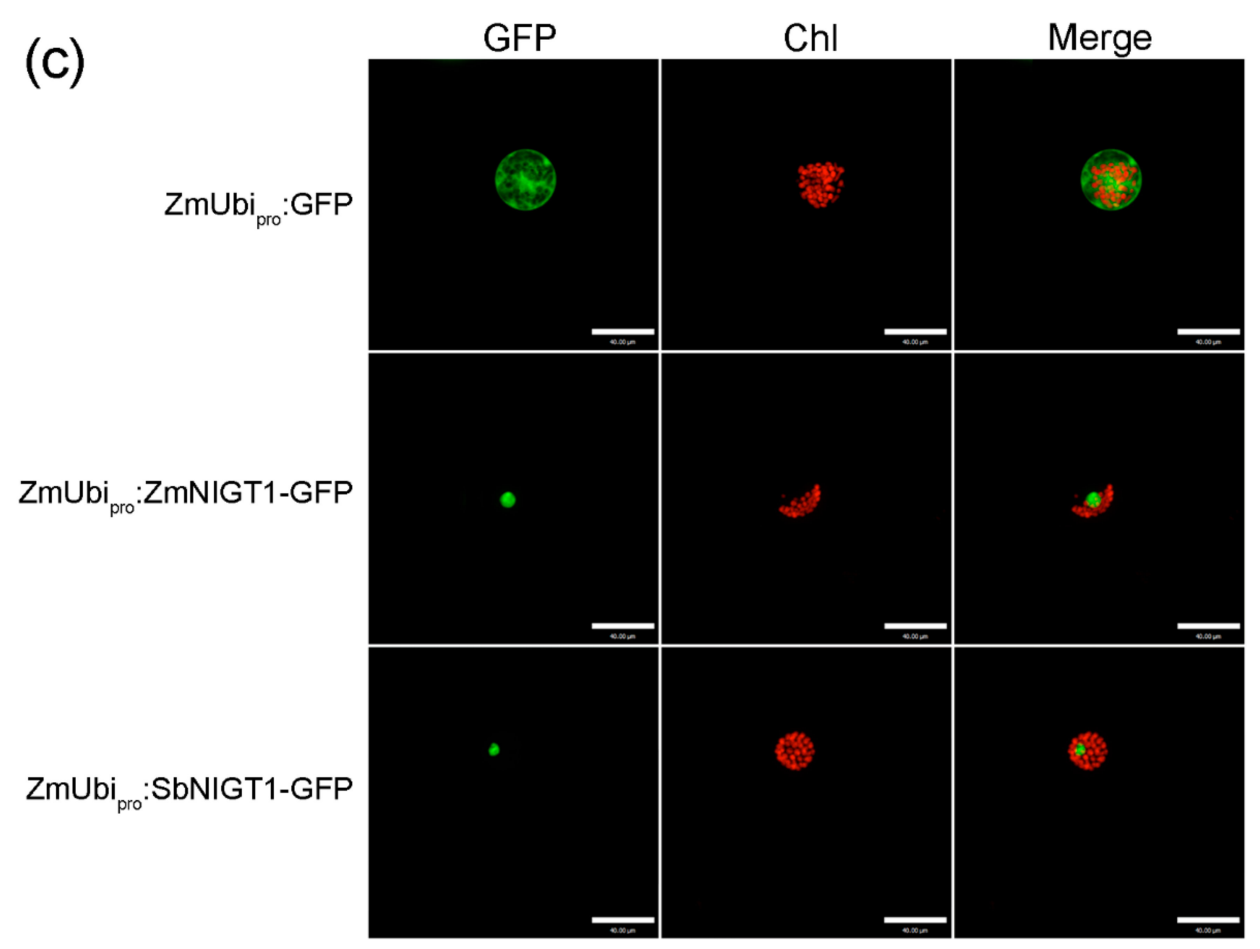
ZmNIGT1 and SbNIGT1 are transcriptional repressors localized in nuclei (15 cm × 20 cm, 300 dpi). (**a**) Phylogenic analysis of ZmNIGT1 and other NIGTs; (**b**) Comparison of GARP motif of ZmNIGT1 protein sequence with other NIGTs; (**c**) Subcellular localization of ZmNIGT1-GFP and SbNIGT1-GFP fusion proteins; (**d**) ZmNIGT1 and SbNIGT1 are transcriptional repressors. Top schematic represents constructs used in transcriptional repressor assay. Quantitative fluorescence-intensity analysis shown in lower bar chart; six repeats were used for two-tailed t test.

To gain insight into the regulation mechanism of hub TFs in the conserved NO_3_^−^-regulatory module, *ZmNIGT1,* and *SbNIGT1* were chosen for further characterization. *ZmNIGT1* and *SbNIGT1* belong to the G2-like subfamily of GARP (Golden2, ARR-B, and Psr1) transcription factors. GARP family members contain a conserved signature motif called the GARP motif (B-motif) that somewhat resembles the MYB-like domain of MYB-related proteins [37]. Time course analysis revealed that *ZmNIGT1* was induced within 0.5 h of nitrate treatment, peaking approximately 3 h later and then decreasing during further nitrate treatment (Figure S3). Phylogenetic analysis indicated close relations between ZmNIGT1 and SbNIGT1 (Figure 5a); sequence alignment showed that they contain a highly conserved GARP motif (Figure 5b). To assess subcellular localization, we fused the CDS of *ZmNIGT1* and *SbNIGT1* to the *GFP* reporter gene and obtained constructs ZmUbi_pro_:ZmNIGT1-GFP and ZmUbi_pro_:SbNIGT1-GFP. Confocal images suggested that the ZmNIGT1 and SbNIGT1 fusion proteins were mainly distributed in the nucleus of maize protoplasts (Figure 5c). To test whether ZmNIGT1 and SbNIGT1 were transcription inhibitors, we generated a reporter 35S-UAS-Luc construct in which *Luc* was placed under the control of 35S-UAS (GAL4 binding site). Constructs 2×35S-G4DBD-ZmNIGT1 and 2×35S-G4DBD-SbNIGT1 were generated as effectors. Co-expression of 2×35S-G4DBD-ZmNIGT1 or 2×35S-G4DBD-SbNIGT1 with 35S-UAS-Luc in tobacco leaves resulted in reduced LUC activity compared with the control (Figure 5d), suggesting that ZmNIGT1 and SbNIGT1 have transcriptional inhibitory activity.

### 2.7. DNA Affinity-Purification Sequencing Identifies Genomic Sites That Are Bound by ZmNIGT1

**Figure 6:**
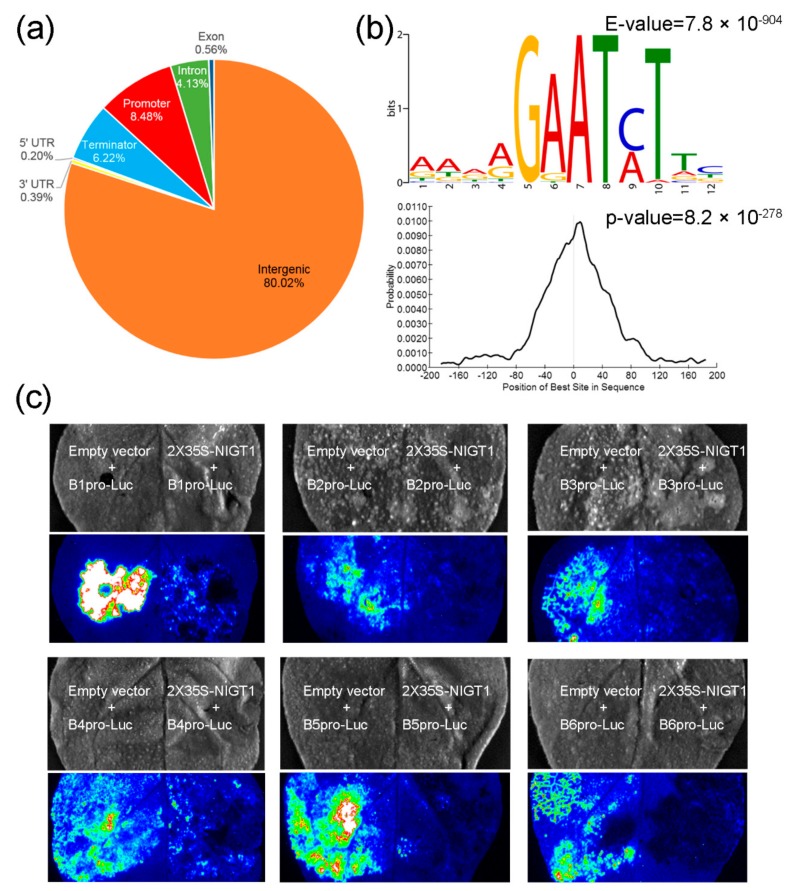
Genome-wide binding profiles of ZmNIGT1 from DNA affinity-purification sequencing (DAP-Seq) analysis (15 cm × 17 cm, 300 dpi). (**a**) Distribution of ZmNIGT1-binding regions in maize genome. Promoter region, −2 kb to transcription start site (TSS); terminator, +2 kb to transcription termination site (TTS); intergenic region, 2 kb upstream of TSS or 2 kb downstream of the TTS. (**b**) ZmNIGT1-binding motif identified by MEME-ChIP in 400 bp flanking sequences around the genic peak summits and density plot of this motif around peak summits. (**c**) Luciferase-activation assay with potential targets of ZmNIGT1. B1, *GRMZM2G123119*; B2, *GRMZM2G028980*; B3, *GRMZM2G140614*; B4, *GRMZM2G133684*; B5, *GRMZM2G159119*; B6, *GRMZM2G025870*.

DNA affinity-purification sequencing (DAP-Seq) was used to identify cis-regulatory elements (CREs) that directly targeted by ZmNIGT1 protein. A total of 19,867 peaks were identified as compared to the negative control (GFP). Among all detected peaks, 80.02% were in the intergenic region, while 8.48% were in the core promoter regions (Figure 6a). The peaks located in the promoter and 5′ UTR of protein-coding genes were used in further analyses, and this generated 1311 peaks that were distributed on 1284 genes (Supplemental Data S7). De novo discovery of the enriched motifs in the binding sites of promoter and 5′ UTR genic regions identified the B-box (GAATC/AT) as the top-scoring motif (E-value = 7.8 × 10^−904^; Figure 6b). This B-box was identical to those of OsNIGT1 and *Arabidopsis* NIGT1-binding sequences (GAATATTC and GAATC) [38,39].

To test whether the B-box mediated the transcriptional regulation of ZmNIGT1, a transient transcription luciferase assay was performed using the promoters of potential ZmNIGT1 targets. Compared to an empty vector, co-expression of 2×35S-ZmNIGT1 with six promoters of targets in tobacco leaves resulted in reduced LUC activity, suggesting that ZmNIGT1 may repress the expression of *Luc* (Figure 6c). *GRMZM2G123119* (B1) encoded an ERF transcription factor that was homologous to *Arabidopsis* ERF1, which mediates crosstalk between ethylene and auxin biosynthesis during primary root elongation [40]. *GRMZM2G028980* (B2) encodes an auxin response factor that regulates auxin-mediated root development [41]. *GRMZM2G159119* (B5) encoded a G2-like transcription factor that was homologous to ZmNIGT1, suggesting that the feedback regulation of NIGT1-clade genes exists in nitrate signaling. *GRMZM2G140614* (B3), *GRMZM2G133684* (B4), and *GRMZM2G025870* (B6) encode proteins that are involved in carbon and nitrogen metabolism.

Among the 1284 potential regulated genes of ZmNIGT1, 537 genes were annotated by agriGO 2.0, enriched with 20 significantly enriched GO terms (Figure S7). According to molecular function, 466 genes were mapped to catalytic activity (GO:0003824). According to cellular component, the 537 genes were mainly related to the intracellular and cell part, organelle, and cytoplasm. Among the potential targets, *GRMZM2G009779* and *GRMZM2G048363* encode phosphate transporters; *GRMZM2G166976* encodes a vacuolar phosphate transporter (VPT); *GRMZM2G146940* encodes a phosphate transporter traffic facilitator (PHF); and *GRMZM5G836174* and *GRMZM2G104942* encode phosphate starvation-induced proteins, suggesting that ZmNIGT1 may be involved in phosphate signaling. In addition, *GRMZM2G444801* and *GRMZM2G444801* encode sulfate transporters, *GRMZM2G047616* encodes potassium transporter, implying that ZmNIGT1 may be involved in various nutrient metabolism. *GRMZM2G051528* encodes a MYB transcription factor that is homologous to AtMYB12, which is involved in gibberellic acid (GA) dependent root growth by regulating flavonol biosynthesis [42].

## 3. Discussion

Nitrogen-use efficiency (NUE) is an important factor in determining crop growth and yield. Understanding the mechanism of crop response to external nitrate supply at the molecular level is crucial work in improving plant NUE. The primary objectives of this study were to move beyond single and dual time point studies and (1) contrast the dynamic NO_3_^−^-regulatory networks across two species with a close evolutionary relationship, maize, and sorghum and (2) identify conserved modules and master regulators from dynamics networks.

### 3.1. Cross-Species Transcriptional Network Analysis Reveals Conserved NO_3_^−^-Regulatory Modules

There were 2920 DEGs in maize and 1975 DEGs in sorghum that responded to nitrate. Most DEGs were consistent with other studies that mainly focused on *Arabidopsis* [31]. Furthermore, a large set of the top 50 consistent and conserved genes in response to nitrate in *Arabidopsis* [31] were identified in the present study (Supplemental Data S1 and S2, Table 2). The DEGs of maize and sorghum were mapped to similar GO terms in biological process and molecular function (Figure 1), although the number of GO terms greatly varied, suggesting that maize and sorghum respond similarly to the nitrate treatments.

Co-expression networks were constructed on the basis of time series of transcriptome data. Despite their genome size and phylogenetic distance, our analysis revealed a set of NO_3_^−^-regulated modules and genes conserved in maize and sorghum (Table 1 and Table 2). The most significant modules were related to nitrate assimilation and carbohydrate metabolism, which can easily be identified in other transcriptome analyses [31,43]. *ZmHPP* ranked in the top positions of the nitrate assimilation-related module (darkturquoise); *At3g47980* (homologous of *ZmHPP*) was a hub gene in a coexpression module of which the top categories were anion transport and response to nitrate [31]. Three G2-like TFs (*NIGT1*/*HRS1*, *HHO2,* and *HHO3*) were found in the top positions of TF ranking [31]. These results implied that the module we constructed in maize may also be conserved with *Arabidopsis*. We also found that part of the nitrate response was missed by previous transcriptional analysis. This contains hormonal signaling such as ethylene and jasmonic acid (Figure S5). Increasing amounts of evidence indicate that hormone biosynthesis, transport, and signaling are partly controlled by nitrate signaling. The relationship between nitrate and hormonal signaling seems to go both ways [44,45]. First, nitrate provision triggers many hormone-related developmental programs that depend on nitrate concentration. Nevertheless, these hormonal pathways can also feedback to nitrate transport, assimilation, and sensing system.

### 3.2. Interaction between Nitrate and Hormone-Mediated Signaling

Four modules that are related to hormonal signaling were conserved in maize and sorghum (Table 1). Ethylene signaling was proved to participate in nitrate-dependent lateral-root (LR) development in *Arabidopsis*. A rapid accumulation of ethylene was detected in roots after transferring seedlings from low to high nitrate, which acts as an inhibitory effect of LR growth by modulating *NRT* expression [46]. *Arabidopsis* AP2/ERF59 can target the GCC boxes in the promoter of *NRT1.8*, and EIN3 can target the EIN3-binding motifs in the promoter of *NRT1.5* [47], suggesting that AP2/ERF TFs are likely to be directly involved in the regulation of *NRT1* expression. Here we identified four syntenic orthologous AP2/ERF TFs in the conserved modules (Table S4), while the regulation mechanism of their involvement in the NO_3_^−^-regulatory networks needs to be further illustrated.

JAs, including jasmonic acid and its derivatives, were involved in regulating plant adaptations to biotic and abiotic stresses in early studies [48]. JAs also have functions in plant developmental events, including primary root development and secondary metabolism [49]. *Arabidopsis* EIL1 interacts with JAZs to positively mediate both JA-dependent primary root growth inhibition and root hair formation [50]. *Arabidopsis* MYC2 and its homologs (MYC3, MYC4, and MYC5) interact with JAZ proteins to mediate the inhibition of primary root growth [49]. The homologs of *MYC2* in maize (*GRMZM2G001930*) and sorghum (*Sobic.007G183200*) were assigned to the conserved lightgreen and violet modules, respectively. JAs are involved in secondary metabolism; JAZs interact with the MYB-bHLH-WDR complexes to regulate JA-mediated anthocyanin accumulation in *Arabidopsis* [51]. MdHIRs repress anthocyanin accumulation by interacting with the MdJAZ2 to inhibit its degradation in apple [52]. Here, five syntenic orthologous JAZs were identified in the conserved modules (Table S4), while the regulation mechanism of their involvement in the NO_3_^−^-regulatory networks also needs to be further illustrated.

### 3.3. Homologs of Two Hub TF Families Conserved in Maize and Sorghum Biologically Validated in Other Plants

Golden 2-like (G2-like) and Lateral Organ Boundaries Domain (LBD) are classes of transcription factors that are specific in plants [37,53], and their roles in NO_3_^−^ signaling were experimentally validated in other plants. NIGT1/HRS1 with HHO1 were identified as critical G2-like transcription factors in *Arabidopsis*. NIGT1 is a transcriptional repressor and significantly enriched in nitrogen starvation response-related genes under nitrogen availability [54]. OsNIGT1 is a nitrate inducible transcriptional repressor, and overexpressing *OsNIGT1* exhibits nitrate response-related phenotypes [38]. *LBD37*/*38*/*39* of *Arabidopsis* act as negative regulators of N-nutrition-related genes [17]. *OsLBD37* is associated with nitrogen signaling, and overexpression of *OsLBD37* significantly affects nitrogen metabolism [55]. Overexpression of *MdLBD13* in the apple callus and *Arabidopsis* inhibits the expression of N-responsive genes and decreases nitrate reductase activity [56]. It is unknown whether those LBDs are directly involved in the expression control of nitrate-inducible genes (*NIA1*, *NIA2*, etc.). Identification of the exact roles of these LBD TFs in regulation of nitrogen-assimilation associated gene expression might uncover a new nitrate signaling pathway.

In the present study, we found that *ZmNIGT1* and *ZmLBD7* acted as hub TFs in the network (Figure 4). *ZmNIGT1* and *ZmLBD7* were assigned in the same module as N-assimilation related genes (*NRT*, *NIA*, *NIR,* and *GLN*), suggesting that they have similar expression pattern. In *Arabidopsis*, N-assimilated related genes were directly regulated by NIN-like protein (NLP) transcription factors that can bind the nitrate-responsive cis-elements (NREs) in the promoter of those genes [57,58]. *ZmNIGT1* and *ZmLBD7* have similar NREs in their promoters (data not shown), implying that they may be regulated by NLPs in response to nitrate. *SbNIGT1* and *SbLBD6* acted as hub TFs in the network that also contained many N-assimilation related genes, implying that they may be regulated by NLPs.

### 3.4. ZmNIGT1 and SbNIGT1 Are Transcriptional Repressors of Nitrate Response

RNA-seq and RT-PCR showed that *ZmNIGT1* was significantly induced by nitrate at 0.5 h, and peaked approximately at 3 h, followed by decreasing during further nitrate treatment (Figure S3). This expression pattern was similar to that of *OsNIGT1* [38]. Transient transcription dual-luciferase assay showed that ZmNIGT1 and SbNIGT1 had transcriptional inhibitory activity, which was consistent with *Arabidopsis* NIGT1 subfamily transcription factors [54,59]. These results suggested that ZmNIGT1 and its homologies in other plants are negative regulators under conditions of high N availability.

DAP-Seq was performed to find ZmNIGT1-bound DNA regions. We identified a set of potential binding sequences, and a B-box (GAATC/AT) element was over-represented (Figure 6). Transient assays showed that ZmNIGT1 could bind six promoters of targets, including genes that are involved in hormone signaling and carbon and nitrogen metabolism. A potential regulated gene (*GRMZM2G159119*) belonged to G2-like transcription factors, suggesting that feedback regulation of NIGT1-clade genes exists in nitrate signaling [58]. Several regulated genes were associated with phosphate transport and starvation responses. *Arabidopsis* NIGT1s shares many features with ZmNIGT1, such as nitrate-inducible expression, transcriptional repressor activity, binding motifs, and auto-negative feedback regulation [54,58,59]. Three genes encode sulfate and potassium transporters, implying that ZmNIGT1 may be involved in the metabolism of various nutrients. Gene Ontology term analysis revealed the significant enrichment of potential regulated genes involved in catalytic activity, as well as genes involved in the intracellular parts and the cytoplasm. Their mechanism of nitrate responses might be conserved among monocots and dicots. Taken together, we propose that ZmNIGT1 is a repressor of nitrate responses that play an important role in coordinating nutrient metabolism and hormone signaling under fluctuating N conditions.

In conclusion, our time-series transcriptome analysis provides useful information for understanding the dynamic NO_3_^−^-mediated molecular mechanism in both species. Cross-species co-expression network analysis revealed several conserved modules that might be associated with the regulation of carbon and nitrogen metabolism, root morphogenesis, and hormonal signaling. In addition, identified hub transcription factors *ZmNIGT1* and *SbNIGT1* in the conserved module might suggest their involvement as key regulators in response to nitrate. In the future, it would be interesting to further functionally characterize *ZmNIGT1* and other nitrate responsive hub transcription factors, which may be potential candidates to improve the NUE of maize and related crops.

## 4. Materials and Methods

### 4.1. Plant Material Preparation

Maize inbred line B73 and sorghum inbred line BTx623 were used in these experiments. Seeds were germinated on filter paper soaked in water for 48 h with a 14/10 h light/dark photoperiod; then, uniform seedlings were transferred into the coconut matrix. The seedlings were regularly fertilized with a deficient-nitrogen (DN) solution to keep growing in a low nitrogen condition until the three-leaf (V3) stage. Experiment groups were treated with a sufficient-nitrogen (SN) solution, and the control groups were treated with non-nitrogen (NN) solution. The roots of maize and sorghum were harvested at 0, 0.5, 1, 1.5, 2, 3, 4, 6, and 8 h after two treatments, frozen in liquid nitrogen, and stored at −80 °C until further analysis. Each sample was a mixture of three plants, and each time point had one biological replicate. A modified Hoagland nutrient solution (5 mM CaCl_2_, 2 mM MgSO_4_, 0.05 mM EDTA-Fe-Na Salt, 0.5 mM KH_2_PO_4_, 50 μM H_3_BO_3_, 10 μM MnCl_2_, 1 μM ZnSO_4_, 0.3 μM CuSO_4_, and 0.5 μM Na_2_MoO_4_) was employed, with 15 mM KNO_3_ as SN solution, 0.15 mM KNO_3_ + 14.85 mM KCl as DN solution, and 15 mM KCl as NN solution.

### 4.2. RNA-Seq Library Construction and Transcriptome Sequencing

Total RNA was isolated from the roots by utilizing RNAprep pure Plant Kit (TIANGEN Biotechnology (Beijing, China) CO., LTD; code No. DP432). One biological replicate consisting of three independent plants was performed. This yielded a set of 36 samples (2 treatments × 2 species × 9 time points). RNA concentration and quality were evaluated using an Agilent 2100 Bioanalyzer (Agilent Technologies, USA). The cDNA libraries were then constructed following Illumina standard protocols and sequenced with an Illumina HiSeq 2500 by Hanyu Biotechnology (Shanghai) Co., Ltd.

### 4.3. Global and Differential Gene Expression Analysis of RNA-Seq Data

After RNA-Seq, sequence reads were trimmed using SolexaQA++ v3.1 [60]. Maize genome assembly B73 (v3) and sorghum genome assembly BTx623 (v3) were downloaded from the Plant Ensembl database (http://plants.ensembl.org/index.html). Cleaned reads were aligned to the reference genome using Tophat v2.0.13 [61]. The number of fragments per kilobase per million mapped reads (FPKM) per gene were calculated using cufflink [62]. Differentially expressed genes (DEGs) were identified using Cuffdiff on the basis of a comparison of the treatment and control with adjusted *p*-value < 0.05, meanwhile the absolute log_2_ of fold change between treatment and control value ≥1. All genes that did not meet the following requirement were omitted from further analysis: genes with average FPKM (values in total 18 samples) lower than 1, or with more than 6 “0 FPKM” (values in the total 18 samples) were considered not expressed; only one of the eight time points showed differential expression. The remaining genes were called DEGs. Gene Ontology (GO) annotation was obtained using web-based agriGO v2.0 [63]. GO enrichment analysis was performed adjusting a *p*-value of < 0.001 as the cutoff and the other parameters used default values.

### 4.4. Gene Network Construction and Visualization

Co-expression networks were constructed using the WGCNA package (v1.67) in R [20]. For analysis accuracy, all genes with an average FPKM lower than 1 or having “0 FPKM” were omitted from further analysis. After filtering, 26,783 maize genes and 19,894 sorghum genes were retained for the WGCNA; gene FPKM was imported into WGCNA. The modules were obtained using step-by-step network construction with default settings, except that the soft threshold was 6 for maize and 8 for sorghum, TOM type was unsigned, min module size was 50, and merge CutHeight was 0.80 for maize and 0.75 for sorghum. The eigengene value was calculated for each module and used to test the association with each time stage. The networks were visualized using Cytoscape v.3.6.1 [64].

### 4.5. Maize-Sorghum Network Comparison

On the basis of orthologous gene pairs, the maize and sorghum co-expression network was compared. Syntenic orthologous gene pairs of the maize and sorghum genomes were downloaded from CoGe: Comparative Genomics (https://genomevolution.org/coge/). A set of 10,445 syntenic gene pairs were conserved between the maize1 subgenome and sorghum, and 6501 syntenic gene pairs were conserved between the maize2 subgenome and sorghum (Supplemental Data S6). To determine whether the overlaps between maize and sorghum modules were significant, Fisher’s exact test was performed. Modules with a *p*-value <1.0 × 10^−5^ were considered as conserved.

### 4.6. Subcellular Localization

The full-length cDNA sequences encoding ZmNIGT1 and SbNIGT1 were amplified from B73 and BTx623 by using the primers described in Supplementary Table S1, and the fragments were subsequently subcloned into the pJIT163-hGFP vector (containing the promoter of maize ubiquitin) for the transient transfection of maize protoplasts. Maize protoplasts were isolated from the leaves of B73 and transformed according to the published protocol with some modifications [65]. The transformed protoplasts were incubated in the dark for 16 to 20 h at approximately 23 °C and then observed using a spinning disk confocal scanning microscope (PerkinElmer (Massachusetts, USA), UltraVIEW).

### 4.7. DAP-Seq and Data Analysis

DAP-Seq was performed as previously described with some modifications [66]. The full-length *ZmNIGT1* cDNA was cloned in pFN19K HaloTag^®^ T7 SP6 Flexi^®^ Vector. HALO-TF fusion proteins were expressed in an in vitro wheat germ system (TNT® T7 Coupled Wheat Germ Extract System). Separately, the genomic DNA (gDNA) was extracted from B73 roots, followed by sonication for 5 min (30S ON/30S OFF) using a Bioruptor Pico (Diangenode, Belgium). Then, the fragmented gDNA was ligated with a truncated NEXTflex^TM^ barcode adapter to create the DNA library. HALO-TFs were immobilized on Magne HALO-Tag beads, washed, and incubated with the DNA library for 1 h (rotated horizontally, 25 °C). After bead washing, DNA was eluted and amplified with indexed TruSeq primers (NEXTflex Rapid DNA-Seq Kit, Bioo Scientific, code No. NOVA-5144-08). GPF was used as mock control. Sequencing was performed on Illumina HiSeq™ 2500 system.

Raw reads from DAP-Seq data were aligned to the B73 (v3) reference genome using the Burrows–Wheeler Aligner (BWA) program with default parameters [67]. Peak calling was performed with model-based analysis of ChIP-Seq (MACS2) [68]. Significant peaks (fold change >2 and q value <10^−2^) with high confidence in both biological replicates were consistent peaks. The significant peaks located in the promoter and 5′ UTR of protein-coding genes were considered to potentially bind ZmNIGT1 and used in further analysis. To search for binding motifs in consistent ZmNIGT1-binding regions, the 400 bp sequence surrounding the peak summit was extracted and submitted to the online version of MEME-ChIP [69].

### 4.8. Transient Expression Assays in Leaves of *Nicotiana benthamiana*

To test whether ZmNIGT1 had transcriptional inhibitory activity, the full-length *ZmNIGT1* cDNA was inserted into vector pMDC83-BD (containing the DNA binding domain of GAL4) to generate effector pMDC83-BD-NIGT1. The reporter vector contained a construct of 35S-UAS-LUC. The effector, reporter and reference constructs were transiently co-expressed in the leaves of *Nicotiana benthamiana* by *Agrobacterium* GV3101 infiltration as described previously [70]. Reporter and reference values were measured by Dual Luciferase Reporter Gene Assay Kit (Beyotime (Shanghai, China) Co., Ltd.; code No. RG027) using the Tecan M200 system.

For generation of the B1pro-Luc, B2pro-Luc, B3pro-Luc, B4pro-Luc, B5pro-Luc and B6pro-Luc reporters, promoters of B1-B6 were amplified from the genomic DNA of B73 using the primer pairs described in Supplementary Table S1, subsequently, they were cloned into vector pGreenII0800-LUC (contain mini 35S). The full-length *ZmNIGT1* cDNA was inserted into vector pMDC83 to generate effector pMDC83-NIGT1. Reporter and effector constructs were transiently co-expressed in the leaves of *Nicotiana benthamiana* by *Agrobacterium* GV3101 infiltration. After incubation in the dark for 24 h and then in the light for 24 h, the leaves were observed using a low-light cooled CCD imaging apparatus (Tanon Biotechnology (Shanghai, China) CO., LTD; 5200 Multi).

### 4.9. RNA Extraction and Real-Time PCR Analysis

The root samples were harvested as described in Section 4.1. Total RNA was isolated from the roots by utilizing RNAprep pure Plant Kit (Tiangen Biotechnology (Beijing, China) CO., LTD; code No. DP432) and reverse transcribed using PrimeScript^TM^ RT reagent Kit with gDNA Eraser (Takara (Beijing, China) Co., Ltd.; code No. RR047A). The primers used for RT-PCR are listed in Supplementary Tables S2 and S3; housekeeping genes *UPF1* of maize (*GRMZM2G163444*) and *UBI1* of sorghum (*Sobic.010G239500*) were used as control. RT-PCR was performed using SYBR^®^ Premix Ex Taq™ II (Takara (Beijing) Co., Ltd.; code No. RR82L) on a LightCycler^®^ 96 Real-Time PCR System (Roche Diagnostics, Switzerland). The relative expression level was calculated using the 2^−ΔΔCT^ method. Every sample had three technical replicates.

## Figures and Tables

**Table 1:** Characterization of conserved modules between maize and sorghum.

Maize Module ^1^	Sorghum Module ^1^	Overlap Number ^2^	*p* Value ^3^	Summarized Function ^4^
lightsteelblue1 (437)	brown4 (437)	28	9.81 × 10^−10^	secondary metabolic process
lightgreen (689)	violet (1822)	153	0.00	response to hormone
midnightblue (719)	violet (1822)	130	0.00	hormone-mediated signaling pathway
salmon (561)	violet (1822)	170	0.00	response to hormone
magenta (1015)	darkseagreen4 (1897)	116	2.07 × 10^−7^	response to organic substance
lightcyan1 (469)	darkorange (2055)	76	4.18 × 10^−10^	cell part
saddlebrown (266)	darkgrey (267)	44	0.00	cell wall biogenesis
sienna3 (528)	darkgrey (267)	50	0.00	plant-type secondary cell wall biogenesis
darkturquoise (550)	grey60 (587)	31	1.93 × 10^−6^	response to nitrate
darkturquoise (550)	orangered4 (192)	28	4.44 × 10^−16^	
lightcyan (409)	darkslateblue (153)	14	1.05 × 10^−7^	photosynthesis
lightcyan (409)	purple (1369)	136	0.00	
pink (4628)	green (1082)	482	0.00	peptide metabolic process
pink (4628)	mediumpurple3 (190)	105	0.00	
pink (4628)	grey60 (587)	169	2.16 × 10^−12^	

^1^ Number of genes in module presented in parentheses. ^2^ Overlap number represents number of syntenic orthologous genes in maize module. ^3^ 0.00 represents *p*-value was too small for R to display. ^4^ Summarized function was according to GO enrichment analysis of genes from maize modules.

**Table 2:** Syntenic orthologous DEGs in darkturquoise module of maize and grey60 and orangered4 modules of sorghum.

Gene ID (Maize)	Gene ID (Sorghum)	Arabi-Symbol ^1^	Arabi-Define
GRMZM2G000739	Sobic.003G229600	UPM1	urophorphyrin methylase 1
GRMZM2G016749	Sobic.006G064100		Protein phosphatase 2C family protein
GRMZM2G021605	Sobic.004G234100		Phosphoglycerate mutase family protein
GRMZM2G033208	Sobic.010G023700		Transketolase
GRMZM2G058760	Sobic.001G067100	RFNR1	root FNR 1
GRMZM2G060079	Sobic.009G130900		oxidoreductase, 2OG-Fe(II) oxygenase family protein
GRMZM2G067402	Sobic.001G449600	AHB1	hemoglobin 1
GRMZM2G076075	Sobic.002G230600	PGI1	phosphoglucose isomerase 1
GRMZM2G076723	Sobic.007G153900	NIA1	nitrate reductase 1
GRMZM2G077054	Sobic.003G258800	GLT1	NADH-dependent glutamate synthase 1
GRMZM2G079381	Sobic.004G309300	NIR1	nitrite reductase 1
GRMZM2G098290	Sobic.006G249400	GLN2	glutamine synthetase 2
GRMZM2G102959	Sobic.004G309300	NIR1	nitrite reductase 1
GRMZM2G105604	Sobic.003G229600	UPM1	urophorphyrin methylase 1
GRMZM2G106190	Sobic.009G154700	FD3	ferredoxin 3
GRMZM2G133684	Sobic.001G134800		Integral membrane HPP family protein
GRMZM2G145914	Sobic.009G130900		oxidoreductase, 2OG-Fe(II) oxygenase family protein
GRMZM2G161245	Sobic.007G137600	MDH	malate dehydrogenase
GRMZM2G177077	Sobic.006G030800	G6PD2	glucose-6-phosphate dehydrogenase 2
GRMZM2G440208	Sobic.005G115600	PGD3	6-phosphogluconate dehydrogenase family protein
GRMZM2G455124	Sobic.003G270800	NRT2.5	nitrate transporter2.5
GRMZM2G173882	Sobic.002G016300		Homeodomain-like superfamily protein
GRMZM5G878558	Sobic.004G312500	NIA1	nitrate reductase 1
GRMZM2G568636	Sobic.007G153900	NIA1	nitrate reductase 1

^1^ Gene symbol of the beat hit *Arabidopsis* gene.

## Supplementary Materials

The following are available online at https://www.mdpi.com/1422-0067/21/4/1445/s1.
