# Supplementary material for: Cross-Species Root Transcriptional Network Analysis Highlights Conserved Modules in Response to Nitrate between Maize and Sorghum
Source: Int J Mol Sci. 2020 Feb 20;21(4):1445. doi: 10.3390/ijms21041445 (PMC7073038; doi:10.3390/ijms21041445)
Supplement: Supplementary file 1 [file ijms-21-01445-s001.zip › ijms-713606-supplementary-final/supplemental files/ijms-713606-supplementary materials.pdf]

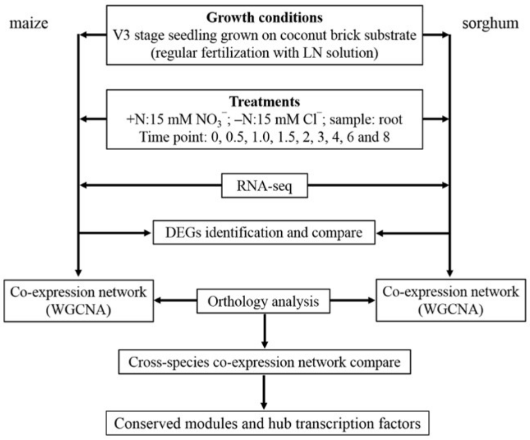

Figure S1. Schematic diagram of the experimental and data-mining approach used in this study.

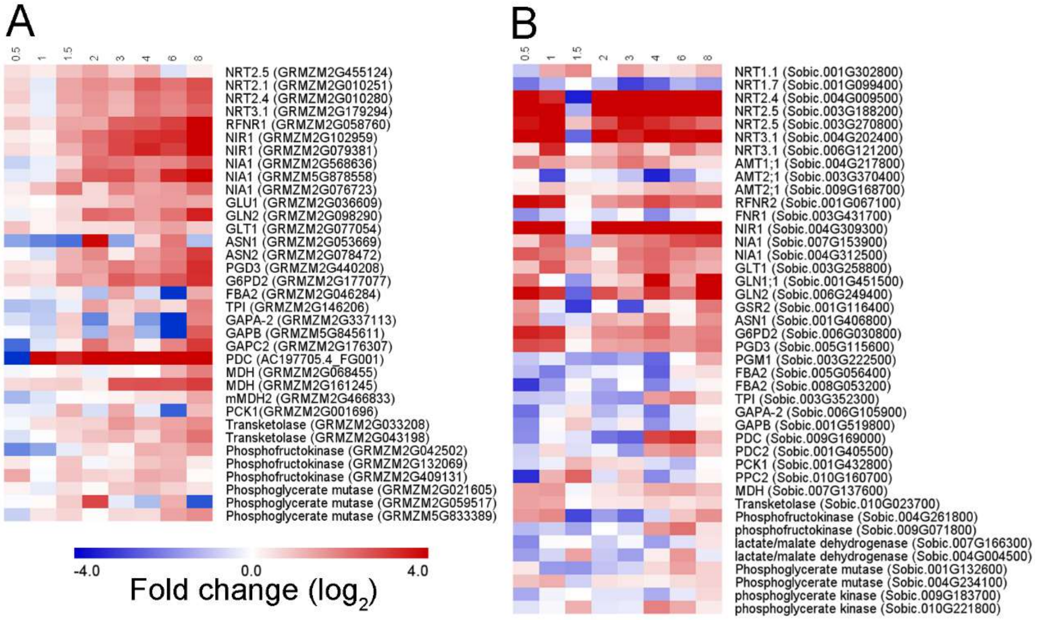

Figure S2. Heat map showing the relative expression of N-assimilation and carbohydrate metabolism related genes.

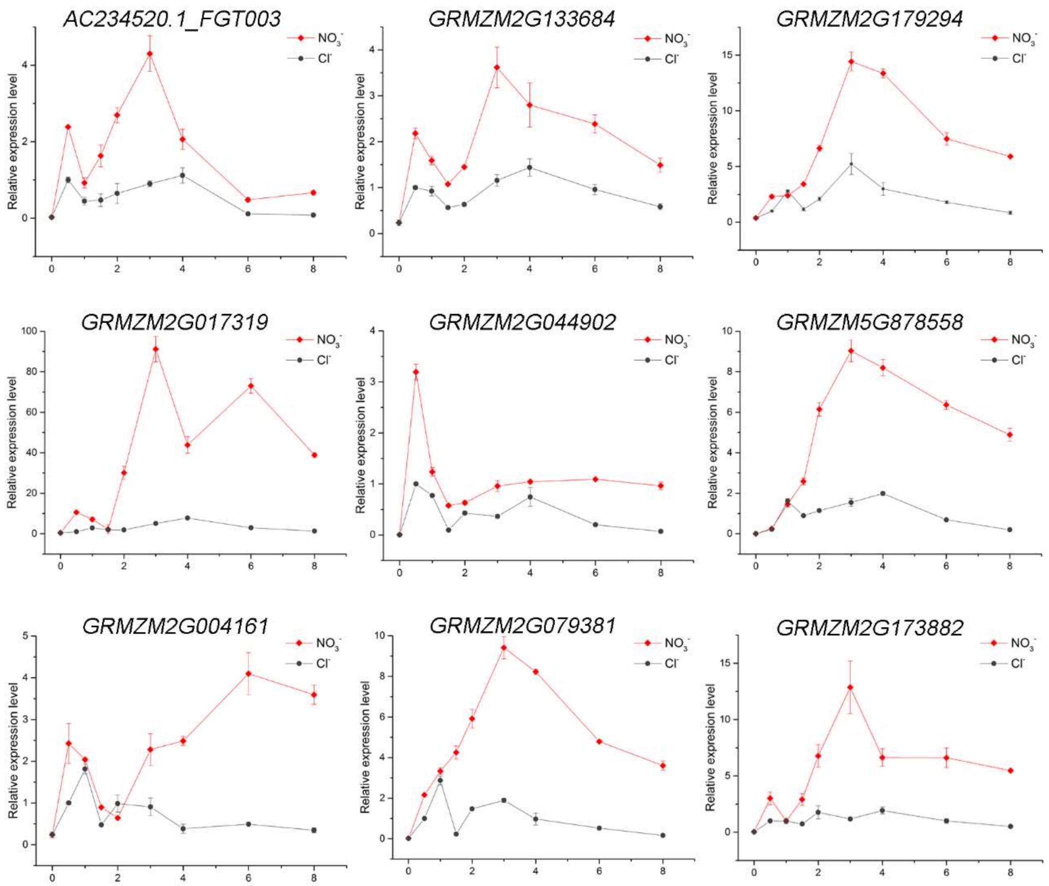

Figure S3. Validation by qRT-PCR of the expression patterns of differentially expressed genes selected from maize. *AC234520.1_FGT003* encodes G2-like transcription factor, *GRMZM2G133684* encodes an integral membrane protein of the HPP family, *GRMZM2G179294* encodes NAR, *GRMZM2G017319* and *GRMZM2G044902* encode LBD transcription factors, *GRMZM5G878558* encodes NIA, *GRMZM2G004161* encodes a BTB protein, *GRMZM2G079381* encodes NRT, GRMZM2G173882 encodes G2-like transcription factor. The error bar indicates the standard deviation of three technical replicates.

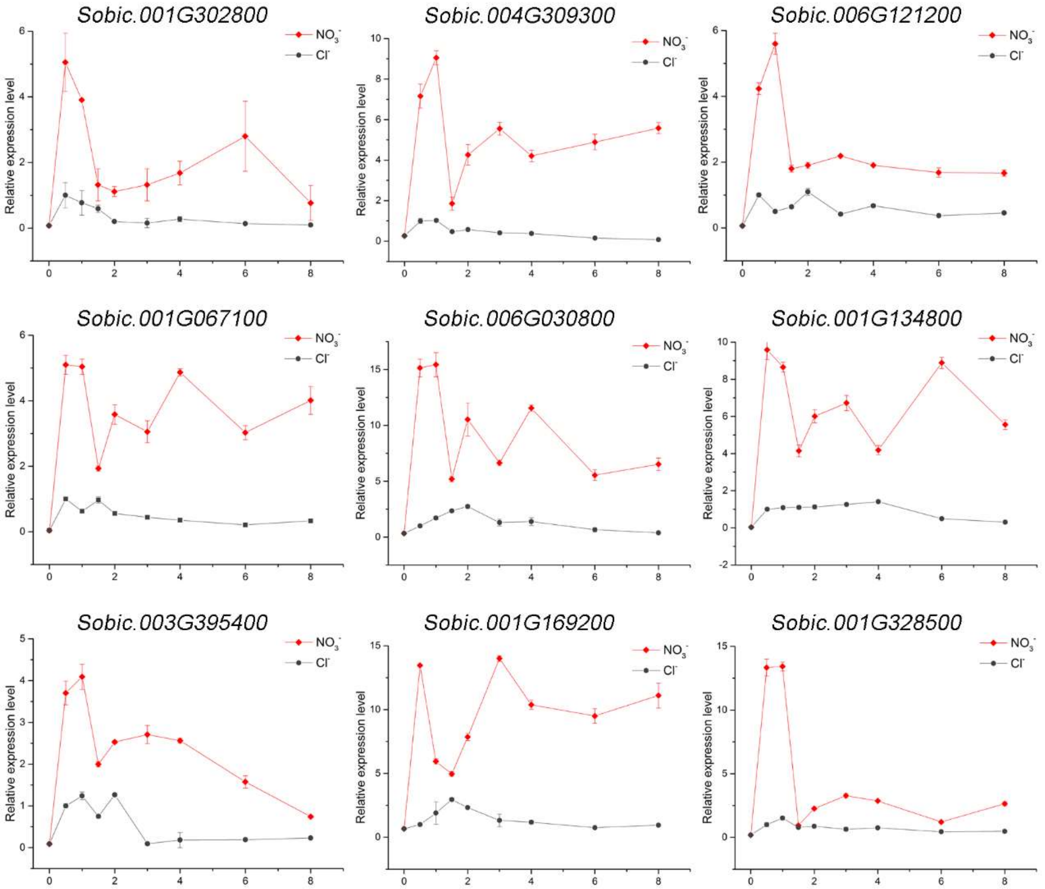

Figure S4. Validation by qRT-PCR of the expression patterns of differentially expressed genes selected from sorghum. *Sobic.001G302800* encodes NRT, *Sobic.001G067100* encodes FNR, *Sobic.001G328500* and *Sobic.001G169200* encode LBD transcription factors, *Sobic.003G395400* encodes a BTB protein, *Sobic.006G121200* encodes NAR, *Sobic.001G134800* encodes an integral membrane protein of the HPP family, *Sobic.004G309300* encodes NIA, *Sobic.006G030800* encodes G6PD. The error bar indicates the standard deviation of three technical replicates.

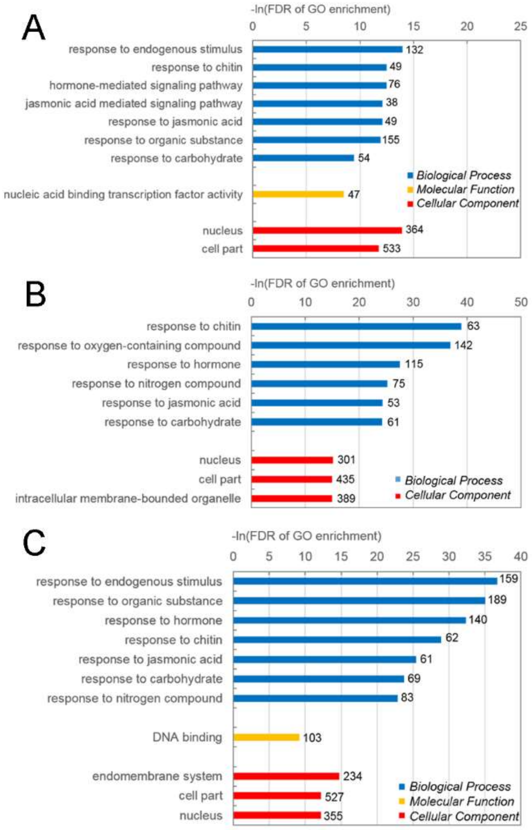

Figure S5. Gene Ontology classification of genes from conserved modules of maize. **A**. midnightblue module; **B**. salmon module; C. lightgreen module. The number next to the bar represent the number of genes in the GO term.

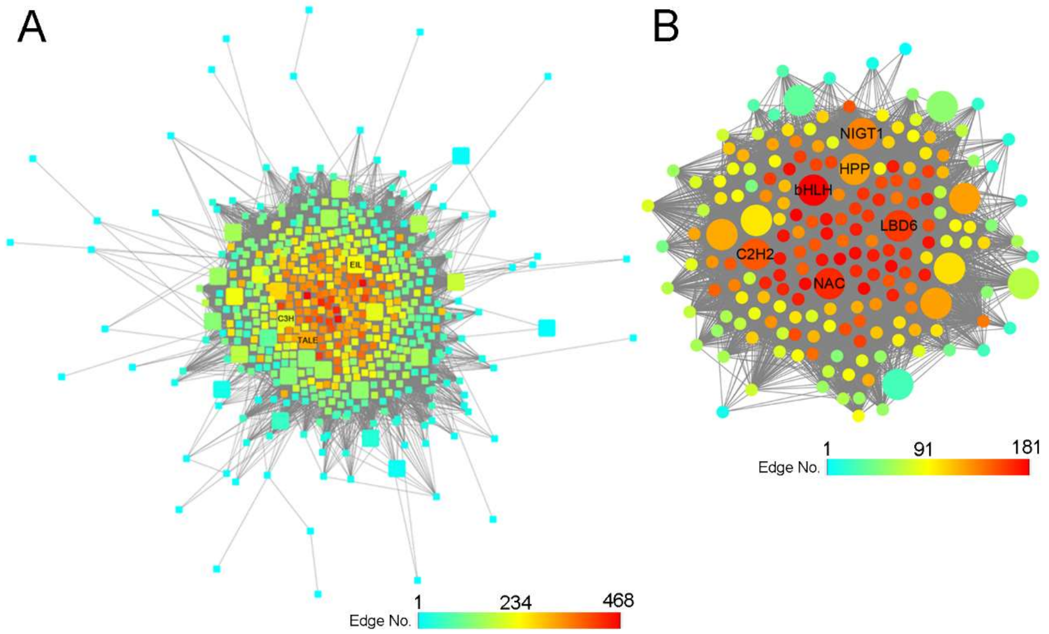

Figure S6. Graphical representation of grey60 and orangered4 module of sorghum. **A**. grey60 module, TALE: Sobic.001G314900, EIL: Sobic.002G247500, C3H: Sobic.004G113700; **B**. orangered4 module,

HPP: Sobic.001G134800, bHLH: Sobic.009G160000, NAC: Sobic.005G064600, LBD6: Sobic.001G328500, C2H2: Sobic.001G020200, NIGT1: Sobic.002G016300.

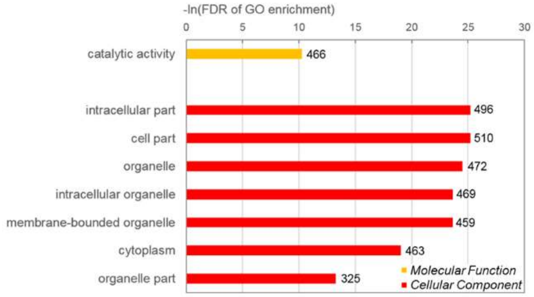

Figure S7. GO enrichment for the potential ZmNIGT1 target genes. The number next to the bar represent the number of genes in the GO term.

Supplementary Table S1 PCR-primers designed for clone and vector construction.

Gene Locus	Forward primer (5'→3')	Reverse primer （5'→3'）
ZmNIGT1	CACACAGGACACCGTAGACAGAT	CAGACAGCTACACTACTCTCAGT
SbNIGT1	ACTCGTGCTTGTTGATTCCTAG	CCTCTTCATCATTTCCAGCCATA
ZmNIGT1-83	*CAGGTCGACTCTAGAGGATCC*GCCACCATGGGGCTGGACGTCGTGGA	*GGGAAATTCGAGCTCGGTACC*TCATTTCCAGCCATAGCTCTCG
ZmNIGT1-BD	*TCGAATCAAGGATCCACGCGT*ATGGGGCTGGACGTCGTGGA	*TTCGAGCTCGGTACCACGCGT*TCATTTCCAGCCATAGCTCTCG
SbNIGT1-BD	*TCGAATCAAGGATCCACGCGT*ATGGAGCTCACCTTCCACGA	*TTCGAGCTCGGTACCACGCGT*TCATTTCCAGCCATAGCTTTC
ZmNIGT1-JIT	*GGTGTTACTTCTGCAAAGCTT*ATGGGGCTGGACGTCGTGGA	*GCCCTTGCTCACCATGGATCC*TTTCCAGCCATAGCTCTCGGAC
SbNIGT1-JIT	*GGTGTTACTTCTGCAAAGCTT*ATGGAGCTCACCTTCCACGA	*GCCCTTGCTCACCATGGATCC*TTTCCAGCCATAGCTTTCTGAC
ZmNIGT1-DAP	*CAGAGCGATAACGCG*ATGGGGCTGGACGTCGTGGA	*AGCCCGAATTCGTTT*TCATTTCCAGCCATAGCTCTCG
SbNIGT1-DAP	*CAGAGCGATAACGCG*ATGGAGCTCACCTTCCACGA	*AGCCCGAATTCGTTT*TCATTTCCAGCCATAGCTTTC
B1pro	*GGTACCGGGCCCCCCCTCGAG*AGCAGTACGCTTCGTTAGGAA	*GCTCTAGAACTAGTGGATCC*CTGGAGTGGATTGGAGTTGAAC
B2pro	*GGTACCGGGCCCCCCCTCGAG*GGACATCAGTACAAGGACAGT	*GCTCTAGAACTAGTGGATCC*CTTGCTTCGCTTTGCTTCTTCT
B3pro	*GGTACCGGGCCCCCCCTCGAG*TTCCATCCATCCTCAATCCATTC	*GCTCTAGAACTAGTGGATCC*CAGGAAGACGAAGGAGAAGAGAT
B4pro	*GGTACCGGGCCCCCCCTCGAG*CGTCAGGATAAAGGTGTCAGTC	*GCTCTAGAACTAGTGGATCC*CCCCTTCTCTTCTCTCCTGTTGA
B5pro	*GGTACCGGGCCCCCCCTCGAG*AGCATTAGCATTCCACTTCTCG	*GCTCTAGAACTAGTGGATCC*GCCCTCCCGGTATTAAATTAAGA
B6pro	*GGTACCGGGCCCCCCCTCGAG*ACCAATAGCCGAATCCGAGATA	*GCTCTAGAACTAGTGGATCC*GCCAATCAACTTGGTCACTAGC

Supplementary Table S2 PCR-primers designed for RT-PCR in maize.

Gene Locus	Forward primer (5'→3')	Reverse primer （5'→3'）
AC234520.1_FGT003	CGAGAAGGCCAAGATGGAGGT	CTCCTTGAGCCAGTCGATCAC
GRMZM2G133684	TCTCCTCCTTACTGAACTCTTGT	CCAAAGCTGCAAACTGTGAAACT
GRMZM2G173882	CCTGGATCTGAGGCACTTCG	CCGCTTCCTCCTTCATCACA
GRMZM2G179294	CATCCACGCCTCTCTCAAGAT	ACCAATGAATCAGCAACGACAG
GRMZM2G017319	CTCTTCCGGTCCCTGCTGTA	CAGTTGCCCGTCCACATGAG
GRMZM2G044902	CAGGCCAAACCAAACCAAACC	GGGACAGATCGTCCAGACAAA
GRMZM5G878558	GAGAGTGGACGAGCATGTCAT	TCCGTTAGCCGCTATTCAGAG
GRMZM2G004161	TAAGCAGCAGGAGCAGGATAA	GGCAGTCTTCACCTTCTTCAC
GRMZM2G079381	CTGGACCGGATGCCCCAACA	CGACGCGGCCGCCCACGAAG

Gene Locus	Forward primer (5'→3')	Reverse primer （5'→3'）
GRMZM2G163444	CACCCGGTTGGCTATGCTGTAC	TGTGCTCCACCAGAAGGCTGAC

Supplementary Table S3 PCR-primers designed for RT-PCR in sorghum.

Gene Locus	Forward primer (5'→3')	Reverse primer （5'→3'）
Sobic.010G239500	CCGTGGTGGCCAGTAAGTTC	GGACTCAACATGGGCTCTGC
Sobic.001G302800	GCCATCAACCTCGTCATCTTC	TTGTGGTAGCAGCATGTATCAG
Sobic.001G067100	TTGCCAATTCAGACAGCCTTC	TTGCCACCACTCCTGTTCTT
Sobic.001G328500	GGACGACGGTAGTAGTATCAGT	GAAGAACTTGGCGACGAAGAC
Sobic.003G395400	TCTAGCCAACAGGAAGCAGAC	CTCACTCCAACCTCTCACTCAT
Sobic.006G121200	AGAGGAGGAAGGACGAGTGA	CAGTGCGTCTTAACCTTGTGA
Sobic.001G134800	CAGATTGGTTGTGCGGCTTT	CATGAATGCTATGCTGGCTGAA
Sobic.001G169200	CGAGAGCTTGAAGACGAAGAT	AAGAACTTGGCGACGAAGAC
Sobic.004G309300	CCTGCTCTCCTCCTACATCAC	AGAAGGTTGAAGCCGAACTTG
Sobic.006G030800	CGGTGAATGGTTGGACAAGAG	CGAGAAGCGAAGGACAGACA

Supplementary Table S4 Syntenic orthologous DEGs in the lightgreen, midnightblue, salmon modules of maize, and violet module of sorghum.

Gene ID (sorghum)	module	Gene ID (maize)	module	arabi-symbol	arabi-defline
Sobic.002G214800	violet	GRMZM2G005954	lightgreen	JAZ1	jasmonate-zim-domain protein 1
Sobic.003G313800	violet	GRMZM2G024476	lightgreen	ATCKX5	cytokinin oxidase 5
Sobic.007G051800	violet	GRMZM2G042895	lightgreen		basic helix-loop-helix (bHLH) DNA-binding superfamily protein
Sobic.004G064500	violet	GRMZM2G062716	lightgreen		Class I glutamine amidotransferase-like superfamily protein
Sobic.001G407600	violet	GRMZM2G074401	lightgreen	FAD8	fatty acid desaturase 8
Sobic.006G089300	violet	GRMZM2G112039	lightgreen		Major facilitator superfamily protein
Sobic.003G102200	violet	GRMZM2G112629	lightgreen		basic helix-loop-helix (bHLH) DNA-binding superfamily protein
Sobic.002G214800	violet	GRMZM2G116614	lightgreen	JAZ1	jasmonate-zim-domain protein 1
Sobic.004G065900	violet	GRMZM2G120320	lightgreen	WRKY40	WRKY DNA-binding protein 40
Sobic.001G407600	violet	GRMZM2G128971	lightgreen	FAD8	fatty acid desaturase 8
Sobic.004G280900	violet	GRMZM2G134023	lightgreen	BRH1	brassinosteroid-responsive RING-H2
Sobic.003G268800	violet	GRMZM2G165099	lightgreen	MAPKKK16	mitogen-activated protein kinase kinase kinase 16
Sobic.009G044400	violet	GRMZM2G166639	lightgreen		2-oxoglutarate (2OG) and Fe(II)-dependent oxygenase superfamily protein
Sobic.001G259600	violet	GRMZM2G173596	lightgreen	JAZ2	TIFY domain/Divergent CCT motif family protein
Sobic.005G109000	violet	GRMZM2G177668	lightgreen	CYP94B3	cytochrome P450, family 94, subfamily B, polypeptide 3
Sobic.006G188700	violet	GRMZM2G397684	lightgreen	BRH1	brassinosteroid-responsive RING-H2
Sobic.008G036000	violet	GRMZM2G424804	lightgreen	CYP94C1	cytochrome P450, family 94, subfamily C, polypeptide 1
Sobic.001G482700	violet	GRMZM5G838098	lightgreen	JAZ1	jasmonate-zim-domain protein 1
Sobic.001G482600	violet	GRMZM2G036351	midnightblue	JAZ2	TIFY domain/Divergent CCT motif family protein
Sobic.002G361500	violet	GRMZM2G053338	midnightblue	AUR32	Auxin-responsive GH3 family protein
Sobic.002G199500	violet	GRMZM2G088819	midnightblue		Calcium-binding EF-hand family protein
Sobic.001G209800	violet	GRMZM2G093951	midnightblue	NHL25	NDR1/HIN1-like 25
Sobic.001G209800	violet	GRMZM2G105987	midnightblue	NHL25	NDR1/HIN1-like 25
Sobic.003G268900	violet	GRMZM2G173965	midnightblue	MAPKKK15	mitogen-activated protein kinase kinase kinase 15
Sobic.001G095700	violet	GRMZM2G301089	midnightblue	BHLH92	basic helix-loop-helix (bHLH) DNA-binding superfamily protein
Sobic.001G100600	violet	GRMZM2G367094	midnightblue		
Sobic.001G482600	violet	GRMZM2G445634	midnightblue	JAZ1	jasmonate-zim-domain protein 1
Sobic.002G335100	violet	GRMZM2G008740	salmon		
Sobic.003G144800	violet	GRMZM2G010016	salmon		
Sobic.007G077100	violet	GRMZM2G020054	salmon	ERF7	ethylene response factor 7
Sobic.009G184400	violet	GRMZM2G020150	salmon	ERF4	ethylene responsive element binding factor 4

Gene ID (sorghum)	module	Gene ID (maize)	module	arabi-symbol	arabi-defline
Sobic.003G268900	violet	GRMZM2G041774	salmon	MAPKKK16	mitogen-activated protein kinase kinase kinase 16
Sobic.009G068900	violet	GRMZM2G057116	salmon	WRKY50	WRKY DNA-binding protein 50
Sobic.003G207900	violet	GRMZM2G066428	salmon		Transmembrane amino acid transporter family protein
Sobic.006G210300	violet	GRMZM2G068967	salmon	ERF7	ethylene response factor 7
Sobic.004G281400	violet	GRMZM2G071277	salmon	BRH1	brassinosteroid-responsive RING-H2
Sobic.001G330100	violet	GRMZM2G113860	salmon	AZF2, ZF2	zinc-finger protein 2
Sobic.009G173300	violet	GRMZM2G125034	salmon		ARM repeat superfamily protein
Sobic.003G146100	violet	GRMZM2G148904	salmon		S-adenosyl-L-methionine-dependent methyltransferases superfamily protein
Sobic.001G138200	violet	GRMZM2G149024	salmon	GATL2	galacturonosyltransferase-like 2
Sobic.003G142300	violet	GRMZM2G158700	salmon	MYB112	myb domain protein 112
Sobic.009G184400	violet	GRMZM2G174347	salmon	ERF9	erf domain protein 9
Sobic.006G273800	violet	GRMZM2G177340	salmon		Polynucleotidyl transferase, ribonuclease H-like superfamily protein
Sobic.001G195100	violet	GRMZM2G181236	salmon	CYP89A6	cytochrome P450, family 87, subfamily A, polypeptide 6
Sobic.001G035000	violet	GRMZM2G361210	salmon		C2H2-type zinc finger family protein
Sobic.003G144800	violet	GRMZM2G454056	salmon		
